# The Effects of Framework Mutations at the Variable Domain Interface on Antibody Affinity Maturation in an HIV-1 Broadly Neutralizing Antibody Lineage

**DOI:** 10.3389/fimmu.2020.01529

**Published:** 2020-07-17

**Authors:** Jeffrey O. Zhou, Hussain A. Zaidi, Therese Ton, Daniela Fera

**Affiliations:** ^1^Department of Chemistry and Biochemistry, Swarthmore College, Swarthmore, PA, United States; ^2^Department of Biology, Swarthmore College, Swarthmore, PA, United States

**Keywords:** human immunodeficiency virus (HIV), antibody, evolution, crystal structure, somatic hypermutation, framework

## Abstract

Understanding affinity maturation of antibodies that can target many variants of HIV-1 is important for vaccine development. While the antigen-binding site of antibodies is known to mutate throughout the co-evolution of antibodies and viruses in infected individuals, the roles of the mutations in the antibody framework region are not well understood. Throughout affinity maturation, the CH103 broadly neutralizing antibody lineage, from an individual designated CH505, altered the orientation of one of its antibody variable domains. The change in orientation was a response to insertions in the variable loop 5 (V5) of the HIV envelope. In this study, we generated CH103 lineage antibody variants in which residues in the variable domain interface were mutated, and measured the binding to both autologous and heterologous HIV-1 envelopes. Our data show that very few mutations in an early intermediate antibody of the lineage can improve binding toward both autologous and heterologous HIV-1 envelopes. We also crystallized an antibody mutant to show that framework mutations alone can result in a shift in relative orientations of the variable domains. Taken together, our results demonstrate the functional importance of residues located outside the antigen-binding site in affinity maturation.

## Introduction

A well-recognized aspect of antibody affinity maturation is the somatic hypermutation (SHM) of the antigen binding regions, i.e., the complementarity determining regions, or CDRs (1). This is even observed in antibodies that target rapidly evolving pathogens, such as HIV-1 and influenza. Antibodies target the viral spike, the envelope (Env) or hemagglutinin (HA), in the case of HIV-1 and flu, respectively (2, 3). In certain cases, antibodies that can neutralize a broad spectrum (>50%) of viral variants can be achieved and these antibodies typically target certain conserved regions of the viral spike. In the case of HIV-1, such broadly neutralizing antibodies (bnAbs) arise in ~10–20% of chronically infected patients after about 5 years of infection. The long time frame is partly due to the fact that the virus rapidly develops escape mutations to avoid antibody recognition (4, 5). This results in bnAbs with high mutation frequencies.

Several bnAb lineages have been identified that target HIV-1 Env or flu HA and they each use different strategies during the affinity maturation process to alter their CDRs to bind the viral spike. In the CAP256-VRC26 bnAb lineage, a disulfide bond was introduced within the heavy chain CDR loop 3 (HCDR3) that rigidified and properly oriented it for binding HIV-1 Env, thereby leading to breadth (6, 7). Rigidification of the HCDR3 was also important in the development of breadth of the CH65–CH67 lineage against flu HA (8). In other cases, structural changes of the paratope were not observed. Instead, there were mutations within the CDRs that introduced residues or rotamers of residues necessary for interacting with Env. This was observed in the DH270 and CH235 antibody lineages where improbable mutations were necessary in interacting with key components of Env and/or Env glycans (9–12). Deletions and/or insertions in the CDRs could also alter the angle of approach of antibodies with Env, allowing them to accommodate glycans on Env, as observed in the PGT121 bnAb lineage (13–16).

While CDRs are responsible for the majority of the direct contacts made with an antigen, SHM does also occur in antibodies outside of their CDRs, in the intervening framework regions (FWRs). While the FWRs help stabilize the antigen-binding site and define the conformations of the CDR loops, the roles of mutations in the FWR are not well-understood. Reports have shown that FWR mutations can be responsible for the thermal destabilization of HIV-1 bnAbs, but not weakly neutralizing HIV-1 antibodies (17, 18). FWR mutations have also been shown to increase the dynamics of antibodies, leading to neutralization breadth (17, 19, 20).

The CH103 bnAb lineage, derived from patient CH505, produced several bnAbs including CH103, which target the CD4 receptor binding site (CD4bs) of the HIV-1 Env (21). Crystal structures of antibody fragments from this lineage showed that the orientation of the heavy chain variable domain (V_H_) changed relative to the light chain variable domain (V_L_) in the transition from intermediate antibody I3.2 to I2 during affinity maturation, potentially through FWR mutations (22). While the CD4bs is relatively conserved, rapid mutations in the nearby Env variable loop 5 (V5) can lead to resistance against CD4bs antibodies (23). In the CH505 patient, Env insertion mutations in V5 occurred throughout virus evolution, which reduced the potency of CH103 lineage bnAb precursors through steric interference (22). In response to these insertions, over the course of affinity maturation the antibody V_L_ domain shifted away from V_H_ and the Env V5 loop to accommodate the V5 loop insertions. Different V_L_ orientations relative to V_H_ were observed in the structures of intermediate antibody I3.2 and of the chimeric antibody, I3.1. These antibodies both contain the V_H_DJ_H_ of I3, but this is paired with either the V_L_J_L_ of the unmutated common ancestor (UCA) in I3.2 or with the V_L_J_L_ of the more mature intermediate antibody I2 in I3.1. Thus, this revealed that the shift in V_L_ was mainly attributed by the identity of the antibody's light chain.

To determine if FWR mutations, specifically ones in the antibody light chain at the V_H_-V_L_ interface, were responsible for the shift observed in the CH103 lineage and breadth development, we performed binding measurements to show that these mutations improved affinity toward an autologous (strain specific) HIV-1 Env and heterologous (different strain) HIV-1 Env with longer V5 loops. We also determined the crystal structure of a mutant, which shows that two residues in the FWR at the V_H_-V_L_ interface could lead to a large part of the observed shift seen in the V_L_J_L_ of the UCA and I2 between intermediate antibodies I3.2 and I2. Thermal melts showed that stability was not altered. Our analysis suggests that few mutations can have a large impact on antigen binding, and that antigen distal FWR mutations occurring during antibody maturation can lead to the structural properties necessary to improve affinity toward an antigen without altering stability.

## Results

### Antibody Interface Mutations Improve Binding to Autologous and Heterologous HIV-1 Envs With Longer V5 Loops

**Figure 1:**
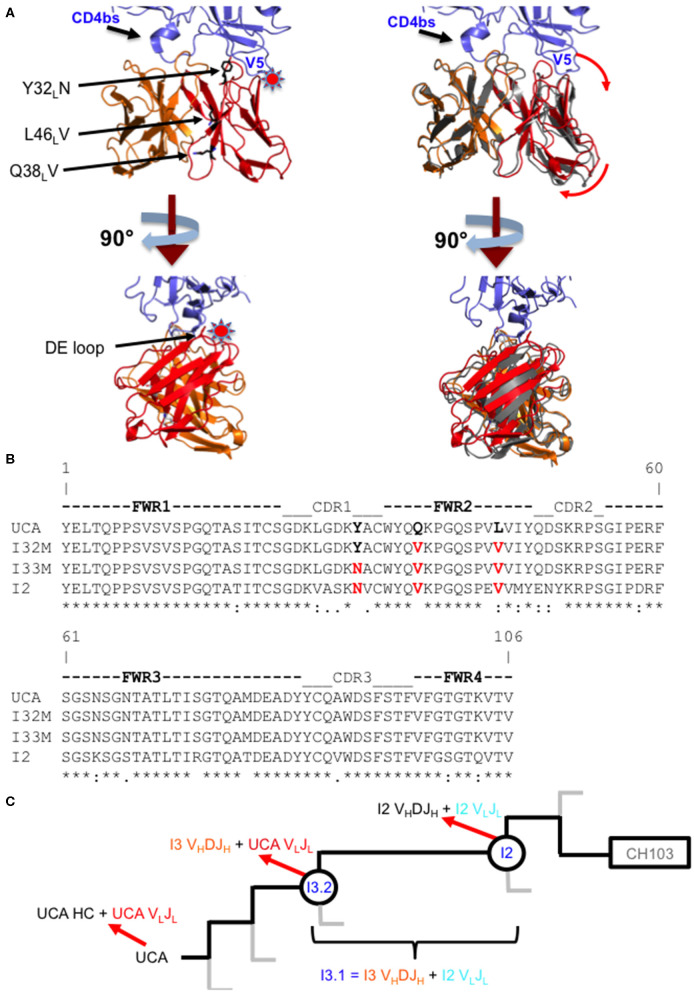
Comparison of wild-type and mutant Fabs. **(A)** Superposition of the I3.2 Fab with the CH103/gp120 outer domain complex. Two orthogonal views of the variable region of the I3.2 Fab (heavy chain V_H_ in orange, light chain V_L_ in red) superposed onto the variable region of the CH103 Fab (gray) in complex with the gp120 outer domain (blue) from ZM176.66 [Protein Data Bank (PDB) ID code 4JAN]. The V5 and CD4 binding site loops of gp120 are marked. A star indicates location of steric clash. Interface mutations introduced in this study are shown as black sticks. Image created in PyMOL. **(B)** Sequence alignment of the variable light chains of I3 wild-type and mutant Fabs. Mutations analyzed in this study are highlighted in red. (*) fully conserved residue, (:) strong conservation of properties, (.) weak conservation of properties, a blank space indicates little to no conservation of properties. **(C)** CH103 bnAb clonal lineage tree. The part of the CH103 lineage under study is shown. Gray lines represent other branches in the lineage. The UCA is shown on the left, the intermediates of interest are circled, and the CH103 bnAb has a rectangle around it. Heavy and light chain compositions are indicated with red arrows. The chimeric antibody is also defined based on its heavy and light chain compositions.

During infection of the CH505 donor, insertion mutations were introduced in the V5 loop of the CH505 HIV-1 envelopes as they evolved (21). While the UCA and early intermediate antibodies had a structure whose V_L_ domain, specifically the antibody DE loop residues 65_L_-67_L_, would clash with V5 loops that were longer than that of the CH505 transmitted founder (T/F) Env, the relative shift in V_L_ with respect to V_H_ appeared to accommodate these V5 loop insertions as well as the longer V5 loops found in heterologous HIV-1 Envs (Figure 1A). As a result, autologous CH505 Envs with longer V5 loops displayed differential binding to different members of the CH103 antibody lineage (i.e., they bound better to later members of the lineage than earlier members), whereas the CH505 T/F Env bound with similar affinity to all CH103 lineage members (Table 1) (22).

**Table 1:** Dissociation constants (*K*_*D*_, in μM) between CH103 lineage Fabs and gp120 core variants.

	**CH505 T/F gp120 core WT**	CH505 T/F gp120 core V5 ETF mutant^*^	**92UG037.8 gp120 core WT**	92UG037.8 gp120 core V5 mutant^**^
I3.2	1.06 ± 0.09	>100	>100	–
I32M	0.98 ± 0.06	15.51 ± 4.11	41.89 ± 8.26	–
I33M	1.86 ± 0.11	3.70 ± 0.18	16.17 ± 2.21	–
I3.1	1.98 ± 0.34	23.74 ± 2.77	3.28 ± 0.83	10.5 ± 0.5^***^
UCA WT	2.40 ± 0.04^***^	NB	NB	52.23 ± 6.37
UCA Q39_H_L	–	–	–	25.10 ± 2.43
UCA S56_H_E	–	–	–	6.98 ± 0.44
UCA Q39_H_L, S56_H_E	–	–	–	2.20 ± 0.30

* *A three residue “ETF” insertion was added to the V5 loop of wild-type CH505 T/F gp120 core*.

** *V5 loop residues “GNINES” of wild-type 92UG037.8 gp120 core were replaced with “KNNT” of CH505 T/F virus Env*.

*** *Published elsewhere (22)*.

*NB denotes no binding detected*.

*Dashes indicate combinations not tested*.

**Figure 2:**
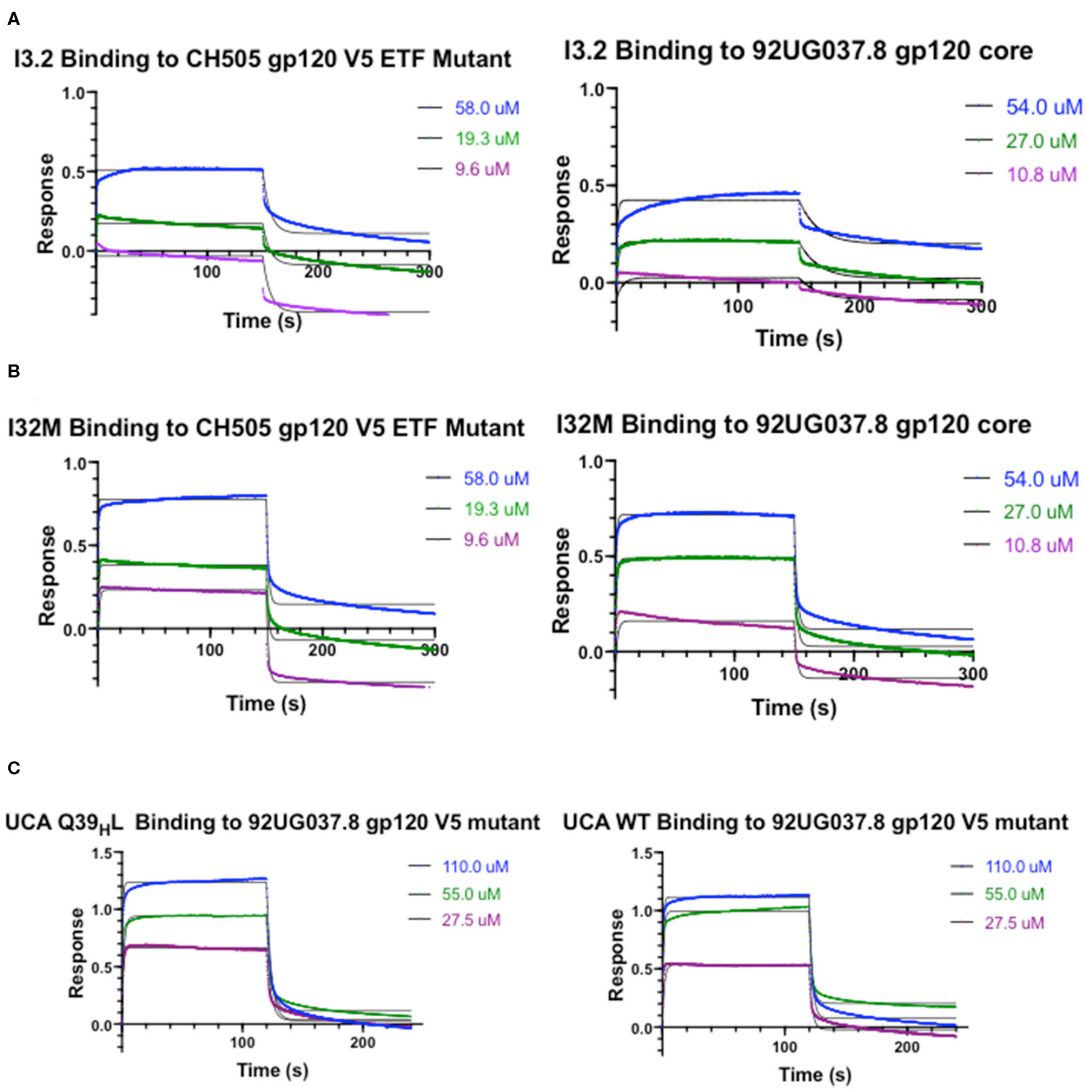
Kinetic Studies by biolayer interferometry. Curves of I3.2 and I32M binding to **(A)** CH505 gp120 core with an “ETF” insertion in the V5 loop and **(B)** 92UG037.8 gp120 core. **(C)** Curves of the UCA and the UCA Q39_H_L mutant binding to 92UG037.8 gp120 core with a V5 mutation. The V5 loop of CH505 T/F Env was grafted in the place of the V5 loop of 92UG037.8 Env. In each case, the Fab was immobilized onto an anti-human Fab-CH1 biosensor, and gp120 constructs were introduced at three or more different concentrations, ranging from low micromolar to high micromolar, depending on the mutant tested.

To assess the role of residues at the V_H_-V_L_ interface on binding Env, we generated an Fab mutant, “I32M”, which consists of two interface mutations in the light chain of I3.2 (i.e., the UCA V_L_J_L_). The light chain mutations we introduced, Q38_L_V and L46_L_V, were based on residues found in the mature members of the lineage (Figures 1B,C). To test binding to HIV-1 Env, we performed biolayer interferometry (BLI) with gp120 core, the monomeric form of the HIV-1 Env ectodomain. Because we wanted to identify differential binding capabilities, we tested binding to an autologous CH505 gp120 T/F core with a three-residue “ETF” V5 loop insertion, which occurred during the natural evolution of the virus in the CH505 patient. I32M bound significantly better than I3.2 to this CH505 gp120 core and with a similar *K*_*D*_ as the chimeric antibody I3.1, which was previously shown to have a V_L_ shifted relative to V_H_ (22) (Figure 2A, Table 1), demonstrating the importance to binding of the two mutations at the V_H_-V_L_ interface, Q38_L_V and L46_L_V. The CH505 T/F wild-type gp120 core did not bind differentially to CH103 lineage members, as expected, nor to our mutant, I32M, which had similar *K*_*D*_ (Table 1). These results suggest that the mutations we introduced did not disrupt binding to Env and that they improve binding to autologous Envs with significantly longer V5 loops.

A hallmark of breadth is the ability of antibodies to bind and neutralize heterologous virus strains. To determine if our I32M mutant could also display improved binding toward heterologous Envs, we tested binding to HIV-1 strain 92UG037.8, whose sequence in the V5 loop differs and is two amino acids longer than that of the CH505 T/F Env. Analysis by BLI shows that I32M binds to this Env with a significantly smaller *K*_*D*_ than its wild-type counterpart I3.2, but not as well as the chimeric antibody I3.1 (Figure 2B, Table 1). These results suggest that two mutations alone in the V_H_-V_L_ interface improve binding to both autologous and heterologous Envs with longer V5 loops, and thereby contribute to breadth against different HIV-1 strains, however, additional light chain mutations may further improve binding to heterologous Envs.

### Few Mutations in the UCA Can Result in Heterologous HIV-1 Env Binding

The UCA is the ancestor of an antibody lineage that is produced against the autologous virus that causes infection. In the UCA of the CH103 lineage, Q39_H_ in the heavy chain forms a reciprocal side-chain hydrogen bond with the light chain Q38_L_ at the V_H_-V_L_ interface (22). Moreover, the I3.2 early intermediate antibody has 18 mutations, only in its heavy chain, when compared to the UCA (Supplementary Figure 1). To determine whether mutations at the V_H_-V_L_ interface can improve binding to the progenitor antibody, we introduced the Q39_H_L mutation in its heavy chain and tested binding by BLI. Since the UCA of the CH103 lineage binds with a similar affinity to the CH505 T/F gp120 core as other members of the lineage and it cannot bind to CH505 or heterologous gp120 cores with longer V5 loops (Table 1), we tested a 92UG037.8 gp120 core whose V5 loop was replaced with a shorter one from the CH505 T/F Env (22). While binding is fairly weak, comparison to the binding of the wild-type UCA shows that the introduction of this single mutation can improve binding by a factor of two (Figure 2C, Table 1). To ensure that the rapid association and dissociation rates observed were not due to bulk shifts at such high gp120 core concentrations, we also tested binding to the VRC01 bnAb, which recognizes the CD4bs but with a different approach angle (24, 25). This bnAb displayed slower on and off rates, distinguishing its binding kinetics from those of CH103 lineage members and their corresponding mutants (Supplementary Table 1, Supplementary Figure 2). This result suggests that the disruption of the V_H_-V_L_ interface Q39_H_-Q38_L_ reciprocal side-chain hydrogen bond is important in affinity maturation and can even improve binding of the earliest antibody lineage member.

Because binding to the UCA Q39_H_L mutant was still relatively weak (*K*_*D*_ = 25.10 μM), we wanted to see if any mutations could further significantly improve binding to the heterologous Env. The published CH103 bnAb-gp120 structure shows that E56_H_ of the CH103 bnAb heavy chain HCDR2 loop engages in hydrogen bonding contacts with the backbone of the conserved gp120 CD4bs (21). Thus, we introduced the S56_H_E mutation into the UCA Q39_H_L mutant and tested binding to the 92UG037.8 gp120 core V5 mutant. The UCA Q39_H_L, S56_H_E double mutant could bind to the 92UG037.8 gp120 core V5 mutant with low micromolar affinity (*K*_*D*_ = 2.20 μM) (Table 1). This data suggests that while the loss of that V_H_-V_L_ interface hydrogen bond is important even for the UCA in gaining breadth against different viruses, the contribution from CDRs is still important.

### V_H_-V_L_ Interface Mutations Alter Relative Orientations of the Antibody Variable Domains

Because Q38_L_ and L46_L_ of the light chain are involved in significant interactions at the V_H_-V_L_ interface and mutating these residues improves binding to Envs, we wanted to determine what structural changes these mutations cause. To aid in crystallization, we added an additional mutation, Y32_L_N, in the light chain of I32M to create “I33M.” Y32_L_N occurs during affinity maturation of the CH103 lineage and N32_L_ first appears in the V_L_J_L_ of intermediate antibody I2 (Figures 1B,C). In later members of the lineage, N32_L_ of the light chain CDR loop 1 (LCDR1) was observed to hydrogen bond with the LCDR2 and HCDR3 loops, stabilizing the conformation of the latter and so we hypothesized the same would happen in I33M.

**Figure 3:**
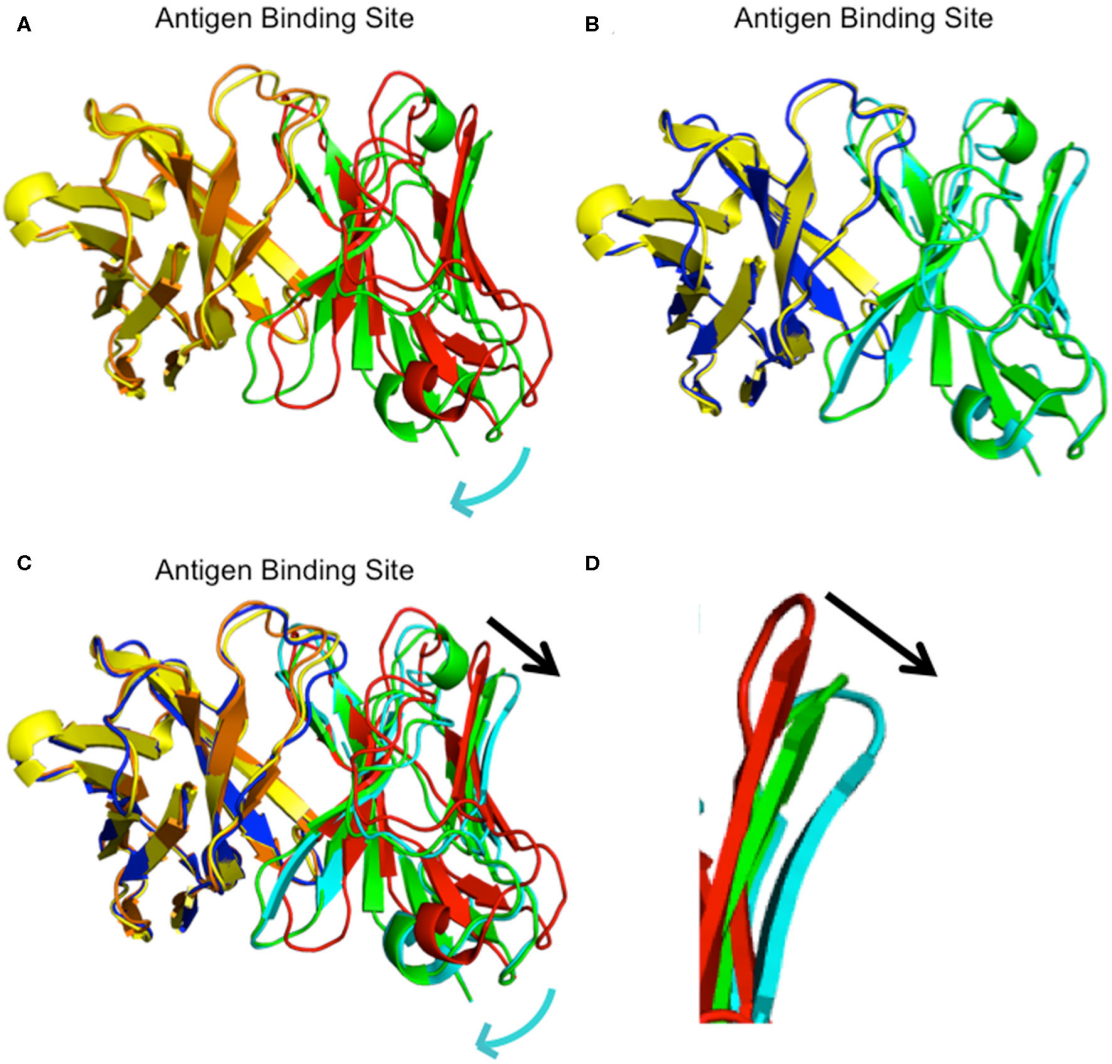
Superpositions of I3.2, I33M, and chimeric I3.1. **(A)** Overall superposition of mutant Fab I33M onto I3.2. The domains are colored: I33M V_H_ (yellow) and V_L_ (green), and I3.2 V_H_ (orange) and V_L_ (red). **(B)** Overall superposition of mutant Fab I33M onto I3.1. The domains are colored: I33M V_H_ (yellow) and V_L_ (green), and I3.1 V_H_ (blue) and V_L_ (cyan). **(C)** Overall superposition of mutant Fab I33M onto I3.2 and I3.1. Domains are colored as in panels **(A,B)**. The constant domains are not shown for clarity. Relative shifts are indicated with a cyan arrow. The black arrow shows the shift in the antibody DE loop. **(D)** Zoomed in view of the DE loops of I3.2 (red), I33M (green), and I3.1 (cyan).

We determined the crystal structure of I33M to a resolution of 2.36 Å (Table 2) and compared it to those of the wild-type intermediate I3.2, the CH103 bnAb, and chimeric I3.1 Fabs (Figure 3, Supplementary Figure 3). The V_H_ of I33M was superposed on that of the wild-type Fabs, since the V_H_ contacts the CD4bs on Env (Figure 1), which is invariant throughout the evolution of the virus and thus expected to bind antibodies in the same way throughout antibody affinity maturation. Superimposed V_H_ domains of I3.2, I33M, and I3.1 demonstrate that I33M has a V_L_ orientation intermediate between that of I3.2 and I3.1 (Figures 3A–C), though it is much more similar to that of I3.1 and to mature members of the antibody lineage (Figure 3B, Supplementary Figure 3A). Additionally, the DE loop of the antibody, from which residues 65_L_-67_L_ clash with the Env V5 loop in the case of I3.2 and the UCA of the lineage, is shifted for I33M in a way that would eliminate the clash, although it is not shifted to the same extent as I3.1 and the CH103 bnAb (Figure 3D, Supplementary Figure 3B).

**Table 2:** Data collection and refinement statistics.

	**I33M**
**Data collection**	
Space group	P6_5_
**Cell dimensions**	
*a, b, c* (Å)	131.6, 131.6, 104.9
α, β, γ (°)	90, 90, 120
Resolution (Å)	50.0–2.36 (2.40–2.36)
Total reflections	249,141
Unique reflections	42,585
Completeness (%)	99.2 (95.7)
*R_*merge*_*	12.6 (97.4)
I/σ(I)	13.0 (1.3)
Multiplicity	5.8 (5.5)
CC(1/2)	0.993 (0.504)
**Refinement**	
Resolution (Å)	47.65–2.36
*R_*work*_*/*R_*free*_* (%)	18.5/22.3
**No. atoms**	
Protein	6,346
Ligand/ion	13
Water	304
Ramachandran favored (%)	97
Ramachandran outliers (%)	1.3
**R.M.S. deviations**	
Bond lengths (Å)	0.008
Bond angles (°)	1.15
***B*****-factors (Å**^**2**^**)**	
Protein	52.70
Ligand/ion	22.30
Water	56.00

* *Values in parentheses are for highest-resolution shell*.

**Figure 4:**
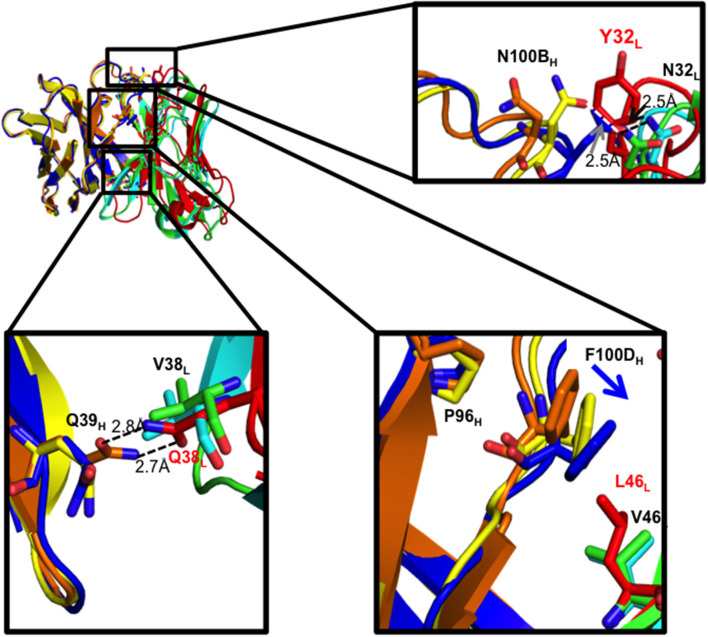
V_H_-V_L_ interface residues and their effects. Zoomed in views of residues that were mutated are shown as sticks for the three Fabs. Domains for the different Fabs are colored as follows: I33M V_H_ (yellow) and V_L_ (green), I3.2 V_H_ (orange) and V_L_ (red), and I3.1 V_H_ (blue) and V_L_ (cyan). Hydrogen bond distances are given.

As expected, the Q38_L_V mutation in I33M disrupted the reciprocal side-chain hydrogen bond interaction with Q39_H_, resulting in more compact hydrophobic packing seen in the mature CH103 lineage Fabs (Figure 4, Supplementary Figure 3). Moreover, in the case of the UCA and I3.2, L46_L_ is involved in hydrophobic packing by bearing on the F100D_H_ ring, which is a bridge between P96_H_ and Y49_L_ at the domain interface. Replacing leucine with the shorter valine at this position in I33M causes F100D_H_ (Y100D_H_ in the case of the CH103 bnAb) to swing closer to Y49_L_ to maintain hydrophobic interactions, while P96_H_ appears unaffected. This is similar to what was observed in I3.1 and the CH103 bnAb (Figure 4, Supplementary Figure 3C) (22). Thus, Q38_L_V and L46_L_V contribute to V_H_-V_L_ packing.

We further analyzed the structures to see if N32_L_ made similar contacts in the different antibodies despite the different extents of orientation shifts of their V_L_ domains relative to V_H_. As with the chimeric I3.1 and mature CH103 bnAb, N32_L_ in I33M could form the same hydrogen bond with the HCDR3 loop, specifically with N100B_H_, which is conserved in all members of the lineage (Figure 4, Supplementary Figures 1, 3C). Of note, the distances between the backbones of light chain residue 32 (Y32_L_ in the case of the early CH103-lineage members UCA and I3.2 and N32L for later members and I33M) and heavy chain residue 100B are very similar for the three Fabs: 6.4 Å for I33M, 6.2 Å for I3.1, and 6.4 Å for I3.2, suggesting that the Y32_L_N mutation does not alter the V_H_-V_L_ domain interface spacing and that the Q38_L_V and L46_L_V mutations are the sole determinants of the observed shift.

To determine what effect Y32_L_N has on binding Envs, we performed BLI with I33M and found that it bound with a stronger affinity to the CH505 gp120 T/F core ETF mutant and 92UG037.8 wild-type gp120 core than I32M but with the same affinity to CH505 gp120 T/F wild-type gp120 core (Table 1). This suggests that the hydrogen bond that N32_L_ makes with the HCDR3 loop, which binds to the CD4bs, contributes to Env binding.

### V_H_-V_L_ Interface Mutations Do Not Alter Antibody Stability

**Figure 5:**
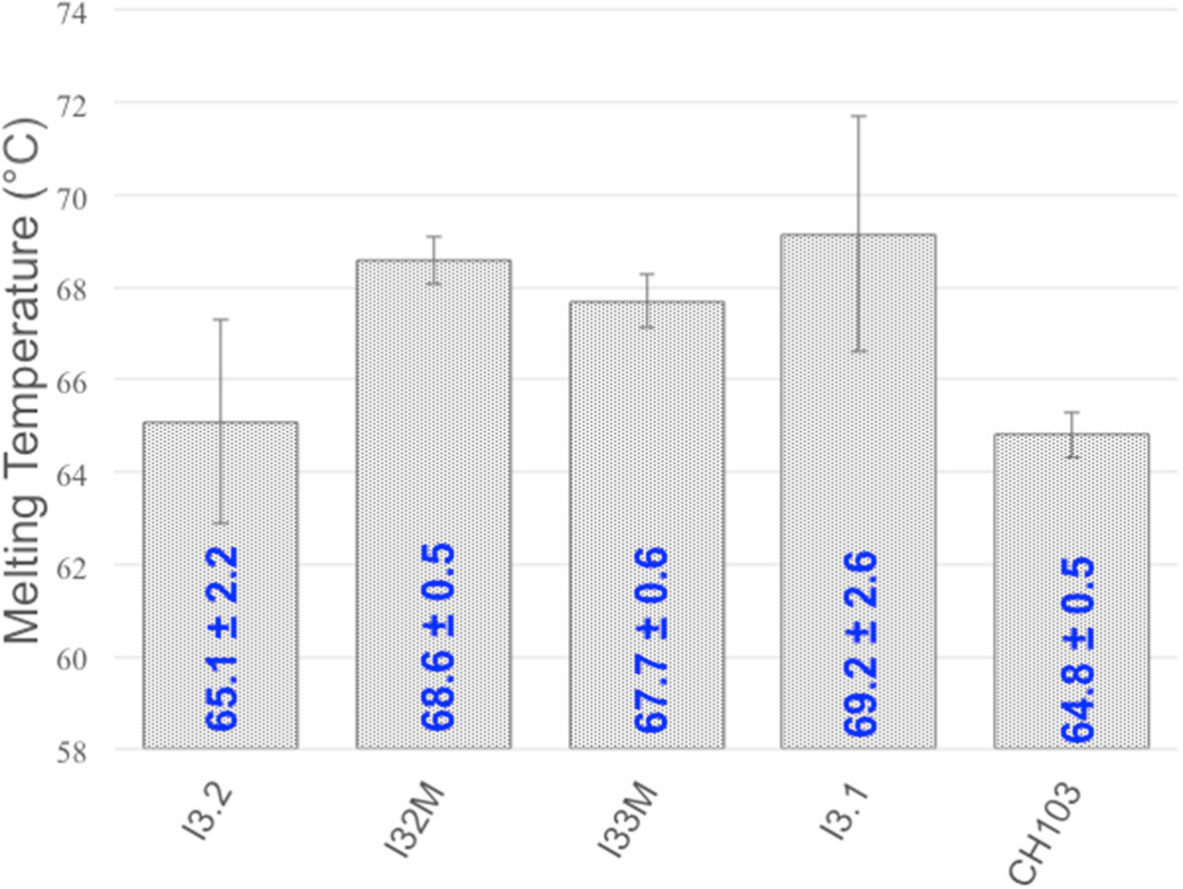
Melting temperatures of Fabs. *T*_*m*_'s of various Fabs and mutants are shown. Standard deviations are from three or more replicates.

To determine what, if any, effects the V_H_-V_L_ interface mutations and structural changes have on thermostability, we measured the melting temperature (*T*_*m*_) of the mutants we generated using circular dichroism thermal melts (Figure 5). *T*_*m*_ values for our mutants are similar and fall within the wide reported range of Fab melting temperatures (65–90°C) (26, 27). Comparison to the melting temperature of I3.2 shows that the introduction of mutations in the light chain at the V_H_-V_L_ interface, or Y32_L_N in the CDR, has no significant effect on thermostability. We also determined the *T*_*m*_ for the CH103 bnAb. CH103 had a similar melting temperature as I3.2, consistent with the reported value of 65.5°C and *T*_*m*_ trend determined by differential scanning calorimetry by others (18).

## Discussion

HIV-1 bnAbs that evolve naturally in infected individuals have high levels of mutations in the CDRs and FWR. While many mutations in the CDR can impact binding to antigen, the roles of the FWR mutations are usually less clear. It has been shown that FWR mutations can be important for the development of breadth (18, 28). In this study, we identified two FWR residues at the V_H_-V_L_ interface, Q38_L_V and L46_L_V, that were important for improving binding to HIV-1 Envs with longer V5 loops. These two mutations alone were sufficient for the significant shift of the orientation of the V_L_ domain with respect to V_H_ over the course of affinity maturation of the CH103 bnAb lineage. Moreover, these mutations did not alter the stability of the antibodies consistent with reports elsewhere (18).

Q38_L_V, distal to the Env binding site, eliminates hydrogen bonding with Q39_H_. Moreover, the Q39_H_L heavy chain mutation first appeared in I2 of the lineage and so I3.2 has a Q39_H_ (Supplementary Figure 1). Our structure of I33M shows that a relative shift in orientation of the domains can occur in the absence of the Q39_H_L mutation, since this mutant only consisted of light chain mutations in I3.2 (Figure 4). Moreover, mutation of just one of these residues alone, which disrupts the variable interface hydrogen bond, is sufficient for improving binding to a heterologous HIV-1 Env (Figure 2C, Table 1), an important feature in the development of breadth. L46_L_V, also within the V_H_-V_L_ interface, alters hydrophobic packing. Thus, the mutations Q38_L_V and L46_L_V in the antibody light chain brought the relative orientation of the V_H_ and V_L_ domains of the antibodies closer to that of the mature members of the CH103 lineage to accommodate Envs with larger V5 loops. Of note, the I32M double mutant bound to the CH505 gp120 core “ETF” V5 loop mutant with a similar dissociation constant (Table 1) as a later intermediate, I2 (22) and the chimeric I3.1. This suggests that all the mutations accrued in late CH103 lineage members may not be necessary for antibody affinity maturation, at least against autologous Envs. These results further demonstrate that it is important to consider more than just the CDRs for vaccine design.

Our work did also identify a few critical CDR mutations that occurred throughout affinity maturation that greatly improved antibody binding to Env. A significant improvement in *K*_*D*_ was observed when we introduced the S56_H_E mutation into the wild-type UCA (~7-fold) or the UCA Q39_H_L mutant (~3-fold; Table 1). Another example of a single CDR mutation enhancing affinity was demonstrated by our I33M mutant, which had the Y32_L_N mutation in the LCDR1. The I33M mutant bound to the 92UG037.8 gp120 core with a ~3-fold lower *K*_*D*_ than that of I32M (Table 1). A likely consequence of the Y32_L_N mutation in the LCDR1 of I33M and other mature CH103-lineage members is the stabilization of the configuration of the HCDR3 loop, important for contacting the conserved CD4bs of the CH505 HIV-1 Env. The HCDR3 loop was disordered in the UCA, but not in later members of the CH103 lineage (22). The hydrogen bonding we observed in I33M between N32_L_ and N100B_H_ (Figure 4) is reminiscent of what is observed in I2 and the bnAbs from this lineage. This illustrates that very few mutations were necessary for the correct configuration of the HCDR3 loop. While FWR mutations have been shown to increase antibody flexibility in some cases (17, 28), results from our crystal structure are consistent with more standard trends observed, i.e., that affinity maturation results in a more rigid antibody structure, specifically in the HCDR3 loop (8, 29–33).

In the CH103 lineage, a significant increase in both heterologous binding and heterologous neutralization was observed during the transition from I3.2 to I2, without much change thereafter (21). Additionally, a shift in the orientation of the V_L_ domain relative to that of the V_H_ was also observed between these two antibodies, and the CH103 bnAb had a similar V_L_ orientation as I2 and the chimeric I3.1 with respect to V_H_ (22). Furthermore, it was shown through structures and binding data, that CH103-lineage Fabs whose V_L_ domain had shifted relative to the V_H_ domain had higher binding affinity toward HIV Envs with longer V5 loops than antibodies from the lineage in which this shift had not yet occurred. Because many other HIV Envs tend to have V5 loops that are longer than that of the CH505 T/F, this suggests there may be a correlation between a shift in V_L_ and heterologous neutralization breadth in the case of the CH103 lineage. Since our I3.2 mutant also had a shift in V_L_, we expect that I32M and I33M have broader neutralization capabilities than the wild-type I3.2, though this has yet to be confirmed experimentally.

A challenge in eliciting antibodies with great breadth via HIV-1 vaccination is the fact that bnAbs take years to develop, requiring high mutation frequencies. Thus, a deep understanding of antibody affinity maturation, paired with virus evolution, is needed to determine key steps in these processes and to identify which stages to target by vaccination. The relative shift in the V_H_ and V_L_ domains was demonstrated to be important and here we show that very few mutations in the antibody variable domain interface are necessary for this structural change. Thus, exposure to an antigen that would lead to such a shift may lead to a more rapid antibody response than one in which many mutations in the antigen combining site are necessary. Our results also have implications for the design of therapeutic antibodies. Alterations at the V_H_-V_L_ interface can alter the antigen-combining site in a favorable way to lead to more potent and broad antibodies.

Complex evolutionary trajectories are followed in different HIV-1 bnAb lineages, therefore, structural analyses at multiple time points during the maturation pathway could reveal new phenomena. Since the shift in orientation of V_L_ relative to V_H_ that we observed in our mutant is similar to but not exactly the same as that observed in the mature CH103 lineage members, there may be additional mutations that would result in a greater shift and should be explored. By comparing available structures of the wild-type, chimeric, and mutant antibodies (22), we find that there are no other mutations in the antibody light chain at the V_H_-V_L_ interface that change identity, rotamers, or amino acid binding partners throughout affinity maturation of this particular lineage. Thus, it is possible that there are mutations in other regions of the FWR or in the CDRs that affect the shift in V_L_ relative to V_H_. Additionally, a high-resolution structure of the CH103 UCA in complex with an Env has not yet been determined. Such a structure is necessary to better understand antibody-virus co-evolution in this lineage as well as properties of HIV-1 Env that triggered the lineage. While the CH103 UCA can only bind the CH505 T/F Env, it binds poorly to an Env stabilized in a closed conformation (data not shown). Our work shows that introducing the S56_H_E mutation in the UCA HCDR2 loop alone significantly improves its binding to a heterologous Env (Table 1). This provides a means for determining an atomic resolution structure of the UCA with Env to provide greater insights for HIV-1 vaccine development.

## Materials and Methods

### Expression and Purification of Fabs

Site-directed mutagenesis was performed using manufacturer's protocols (Stratagene) to introduce mutations into the UCA heavy and light chains. Fabs were expressed in HEK293F suspension adapted cells via transient transfection using linear polyethylenimine (PEI) following the manufacturer's suggested protocol. For 200 mL of suspension cell culture, 100 μg of total plasmid was used. After 5 days of expression, the supernatant was clarified by centrifugation. Each His-tagged CH103-lineage Fab was loaded onto Ni-NTA superflow resin (Qiagen) pre-equilibrated with Buffer A (10 mM Tris, pH 7.5, 200 mM NaCl), washed with Buffer A then Buffer A + 10 mM imidazole, and finally, eluted with Buffer A + 350mM imidazole. Each Fab was then purified by gel filtration chromatography using a Superdex^TM^ 200 Increase 10/300 GL column (GE Healthcare) in buffer of 10 mM Tris, pH 7.5, 100 mM NaCl. Fractions of interest were further concentrated.

Plasmids containing the CMVR VRC01 Ig heavy and light chains were obtained from the NIH Aids Reagents Program. The protein was expressed in 293F cells as described for Fabs above. The clarified supernatant was diluted twofold using 1× PBS buffer and purified using protein A agarose resin (Pierce), according to manufacturer's protocols. The eluent was concentrated and further purified by gel filtration chromatography in buffer of 10 mM Tris, pH 7.5, 100 mM NaCl using a Superdex^TM^ 200 Increase 10/300 GL column (GE Healthcare). The VRC01 Fab was obtained by digesting the Ig using papain (Pierce) and by running the digested products through protein A resin (Pierce) according to manufacturer's protocol. The Fab was then purified by gel filtration chromatography again as described above.

### Expression and Purification of HIV-1 Env gp120s

The codon-optimized synthetic constructs of the CH505 T/F HIV-1 subtype C gp120 containing amino acid (a.a.) 41–492 (HXB2 numbering) ΔV123 (core) was described previously (22). Briefly, the same pVRC-8400 expression vector as described for Fabs was used and the construct contained an N-terminal 6 × -histidine tag. The expression construct also contained a leader sequence encoding the tissue plasminogen activator signal sequence. The IRES-puro vector containing 92UG037.8 HIV-1 subtype A gp120 a.a. residues 1–492 (HXB2 numbering) ΔV123 (core) with a 6 × -histidine tag inserted between residues 40 and 41 and V5 mutations was also described previously (22). Recombinant Env glycoproteins were expressed in HEK 293T cells and purified as described for His-tagged Fabs.

### Bio-layer Interferometry

Kinetic measurements of Fab binding to HIV-1 Env gp120 cores were carried out using the fortéBIO BLItz instrument using the same buffer all of our proteins were purified in (10 mM Tris, pH 7.5, 100mM NaCl) to minimize bulk shift effects. Fabs at concentration 0.2 mg/mL were immobilized on Dip and Read^TM^ Anti-Human Fab-CH1 second Generation Biosensors until saturation was reached (3 min). Env constructs were tested at concentrations ranging from 9.6 to 110 μM, depending on the particular construct used and its solubility, as well as based on its binding affinity to each Fab tested. The CH505 T/F gp120 wild-type core was tested at concentrations of 6.8, 22.0, and 66.0 μM, the CH505 T/F gp120 core ETF mutant was tested at concentrations 9.6, 19.3, and 58.0 μM, the 92UG037.8 gp120 core was tested at concentrations 10.8, 27, and 54.0 μM, and the 92UG037.8 gp120 core V5 mutant was tested at concentrations 27.5, 55.0, and 110.0 μM. Association and dissociation were each monitored for 2–2.5 min. Step corrections, at the start of association and at the start of dissociation, were performed manually using Microsoft Excel. Analyses were then performed using a global fit of at least three measurements using nonlinear regression curve fitting using the Graphpad Prism software, version 8.

### Circular Dichroism

CD spectra were recorded over the range of 260–210 nm to confirm the presence of a β-sheet signal at 216 nm (34) using an AVIV Model 435 Circular Dichroism Spectrometer. Fabs were loaded into 700 μL quartz crystal cuvettes at a concentration of 0.02 mg/mL in PBS buffer. Thermal melts were done from 20 to 90°C. CD signals at 216 nm were monitored every 0.5°C as the temperature of the samples increased at a ramp rate of 0.5°C/min. The samples were allowed to equilibrate for 5 s at each temperature before CD signals were recorded at an averaging time of 15 s. Data was analyzed with OriginLab using signal smoothing and assuming a two-state model with constant Δ*H* (35). In order to obtain the best fits, the approximated starting and ending baselines (which were assumed to be linear), melting temperature, and enthalpy of unfolding were adjusted. Curves were fit to a chi-squared tolerance value of 1 × 10^−9^.

### Crystallization of CH103 Lineage Fab Mutants

I33M was concentrated to 11.24 mg/mL prior to crystallization. I33M crystallized in 96-well-tray format, set up with a TTPLabTech Mosquito® HTS in 1.5 M AmSO_4_, 100 mM Hepes, pH 7.0, 5% PEG 400 and was optimized in 24-well-format in which the pH of the solution was adjusted to 7.23.

### Data Collection, Structure Determination, and Refinement

X-ray diffraction data was collected using beamlines at the Advanced Photon Source at Argonne National Laboratory. I33M data were collected at beam line 24-ID-E using a 0.98 Å wavelength. Data set from an individual crystal was processed with HKL2000 (36). Molecular replacement calculations for the Fab were carried out with PHASER (37) using published I3.2 [Protein Data Bank (PDB) ID 4QHL] as the search model. The Fab was broken up into the variable and constant domains in order to perform two separate searches. I33M contained two molecules in the asymmetric subunit.

Refinement was carried out with PHENIX (38), and all model modifications were carried out with Coot (39) and PyMOL. During refinement, maps were generated from combinations of positional, group B-factor, and TLS (translation/libration/screw) refinement algorithms. All refinements used secondary structure and NCS restraints. Structure validations were performed periodically during refinement using the MolProbity server (40). The final refinement statistics are summarized in Table 2.

### Protein Structure Analysis and Graphical Representations

The structures analyzed in this study were superposed by least squares fitting in Coot.

## Data Availability Statement

The datasets generated for this study can be found in the Protein Data Bank accession code 6VPY.

## Author Contributions

JZ and DF coordinated and designed the study, analyzed and evaluated the data, and wrote and edited the manuscript and figures. JZ, HZ, TT, and DF performed experiments: TT, JZ, and DF expressed and purified proteins. TT and HZ performed BLI experiments. JZ collected CD spectra and performed thermal melts. JZ and DF crystallized proteins and determined structures. All authors contributed to the article and approved the submitted version.

## Conflict of Interest

The authors declare that the research was conducted in the absence of any commercial or financial relationships that could be construed as a potential conflict of interest.
