# Corrigendum: The Effects of Framework Mutations at the Variable Domain Interface on Antibody Affinity Maturation in an HIV-1 Broadly Neutralizing Antibody Lineage

**DOI:** 10.3389/fimmu.2020.620518

**Published:** 2020-11-18

**Authors:** Jeffrey O. Zhou, Hussain A. Zaidi, Therese Ton, Daniela Fera

**Affiliations:** ^1^Department of Chemistry and Biochemistry, Swarthmore College, Swarthmore, PA, United States; ^2^Department of Biology, Swarthmore College, Swarthmore, PA, United States

**Keywords:** human immunodeficiency virus, antibody, evolution, crystal structure, somatic hypermutation, framework

In the original article, we neglected to include the funder NIGMS from the NIH, P30 GM124165 to NE-CAT beamlines; the funder NIH-ORIP HEI, S10OD021527 to the Eiger 16M detector on the 24-ID-E beam line; and the funder US DOE, DE-AC02-06CH11357 to APS. The corrected funding section appears below:

JZ was supported by the Meyer Davidson ’57 Summer Research Fellowship Grant. DF acknowledges support from the Mathilde Krim Fellowship in Basic Biomedical Research (109502-61-RKVA) from amfAR and Swarthmore College Startup and Faculty Research Funds. This work is based upon research conducted at NE-CAT beamlines, which are funded by the NIGMS from the NIH (P30 GM124165). The Eiger 16M detector on the 24-ID-E beam line is funded by a NIH-ORIP HEI grant (S10OD021527). This research used resources of the Advanced Photon Source, a U.S. Department of Energy (DOE) Office of Science User Facility operated for the DOE Office of Science by Argonne National Laboratory under Contract No. DE-AC02-06CH11357.

The authors apologize for this error and state that this does not change the scientific conclusions of the article in any way. The original article has been updated.

