# Supplementary material for: The Effects of Framework Mutations at the Variable Domain Interface on Antibody Affinity Maturation in an HIV-1 Broadly Neutralizing Antibody Lineage
Source: Front Immunol. 2020 Jul 17;11:1529. doi: 10.3389/fimmu.2020.01529 (PMC7379371; doi:10.3389/fimmu.2020.01529)
Supplement: Supplementary file 1 [file Data_Sheet_1.pdf]

Supplementary Table 1. Binding of CH103 lineage Fabs to gp120 core variants.

	**CH505 T/F gp120 core WT**		
	K_D (μM)	k_{on} ($x10^3\ M^{-1}s^{-1}$)	k_{off} ($x10^{-1}\ s^{-1}$)
**I3.2**	1.06 ± 0.09	21.32 ± 0.59	0.24 ± 0.004
**I32M**	0.98 ± 0.06	14.34 ± 0.57	0.14 ± 0.003
**I33M**	1.86 ± 0.11	29.61 ± 1.12	0.55 ± 0.009
**I3.1**	1.98 ± 0.34	24.25 ± 1.42	0.25 ± 0.005
**UCA WT*** **	2.40 ± 0.04	87.16 ± 2.84	2.10 ± 0.06
**VRC01*** **	1.16 ± 0.03	1.13 ± 0.02	$1.30 \pm 0.017 \times 10^{-2}$
	**CH505 T/F gp120 core V5 ETF mutant***		
	K_D (μM)	k_{on} ($x10^3\ M^{-1}s^{-1}$)	k_{off} ($x10^{-1}\ s^{-1}$)
**I3.2**	>100	---	---
**I32M**	15.51 ± 4.11	18.63 ± 8.90	2.52 ± 0.62
**I33M**	3.70 ± 0.18	34.69 ± 0.78	1.28 ± 0.09
**I3.1**	23.74 ± 2.77	12.98 ± 1.14	3.05 ± 0.09
**UCA WT**	NB	NB	NB
**VRC01*** **	1.44 ± 0.04	0.40 ± 0.01	$5.75 \pm 0.11 \times 10^{-3}$
	**92UG037.8 gp120 core WT**		
	K_D (μM)	k_{on} ($x10^3\ M^{-1}s^{-1}$)	k_{off} ($x10^{-1}\ s^{-1}$)
**I3.2**	>100	---	---
**I32M**	41.89 ± 8.26	10.66 ± 1.86	4.40 ± 0.29
**I33M**	16.17 ± 2.21	4.87 ± 0.49	0.78 ± 0.03
**I3.1**	3.28 ± 0.83	24.67 ± 1.18	0.95 ± 0.02
**UCA WT**	NB	NB	NB
**VRC01**	8.11 ± 0.88	0.53 ± 0.03	$4.29 \pm 0.21 \times 10^{-2}$
	**92UG037.8 gp120 core V5 mutant****		
	K_D (μM)	k_{on} ($x10^3\ M^{-1}s^{-1}$)	k_{off} ($x10^{-1}\ s^{-1}$)
**UCA WT**	52.23 ± 6.37	81.22 ± 0.71	4.20 ± 0.15
**UCA Q39$_H$L**	25.10 ± 2.43	11.03 ± 0.76	2.61 ± 0.07
**UCA S56$_H$E**	6.98 ± 0.44	4.39 ± 0.21	0.31 ± 0.01
**UCA Q39$_H$L, S56$_H$E**	2.20 ± 0.30	7.94 ± 0.55	0.25 ± 0.04
**VRC01**	2.27 ± 0.16	1.06 ± 0.03	$2.41 \pm 0.09 \times 10^{-2}$

*A three residue "ETF" insertion was added to the V5 loop of wild-type CH505 T/F gp120 core.
**V5 loop residues "GNINES" of wild-type 92UG037.8 gp120 core were replaced with "KNNT" of CH505 T/F virus Env.
***Published elsewhere [22].
NB denotes no binding detected.
Dashes indicate combinations not tested.

```
           1                                                          60
           |                                                          |
           -----------FWR1---------------CDR1------FWR2----      CDR2
UCA   VH   QVQLQESGPGLVKPSETLSLTCTVSGGSISSYYWSWIRQPPGKGLEWIGYIYYSGSTNYN
I3    VH   -------------S--------------MGG-----L--S-V---------FHT-H----
I2    VH   ----------V--S--------------MGGT----L-LS-----------FHT-H----
CH103 VH   ----------V--S--------------MGGT----L-LS-----------FHT-E---S

                                82abc          100         101       112
                                ||||            |           |          |
           ---------------------FWR3--------------    CDR3    --FWR4----
UCA   VH   PSLKSRVTISVDTSKNQFSLKLSSVTAADTAVYYCASLPRGQLVNAYFDYWGQGTLVTVS
I3    VH   ---E----V-----E-----R-R-----------------------F--N---------A
I2    VH   ---EG—S-------ED----R-R----------F--------------RN--R----S-T
CH103 VH   ----G—S-------ED----R-R----------F--------------RN--R-S----T
```

Supplementary Figure 1. Sequence alignments of CH103 Fab heavy chain sequences. Complementarity determining region (CDR) loops and the framework region (FWR) for each chain are indicated. Conserved residues with respect to the UCA are marked by a dash. Sites mutated in this study are indicated with a red arrow.

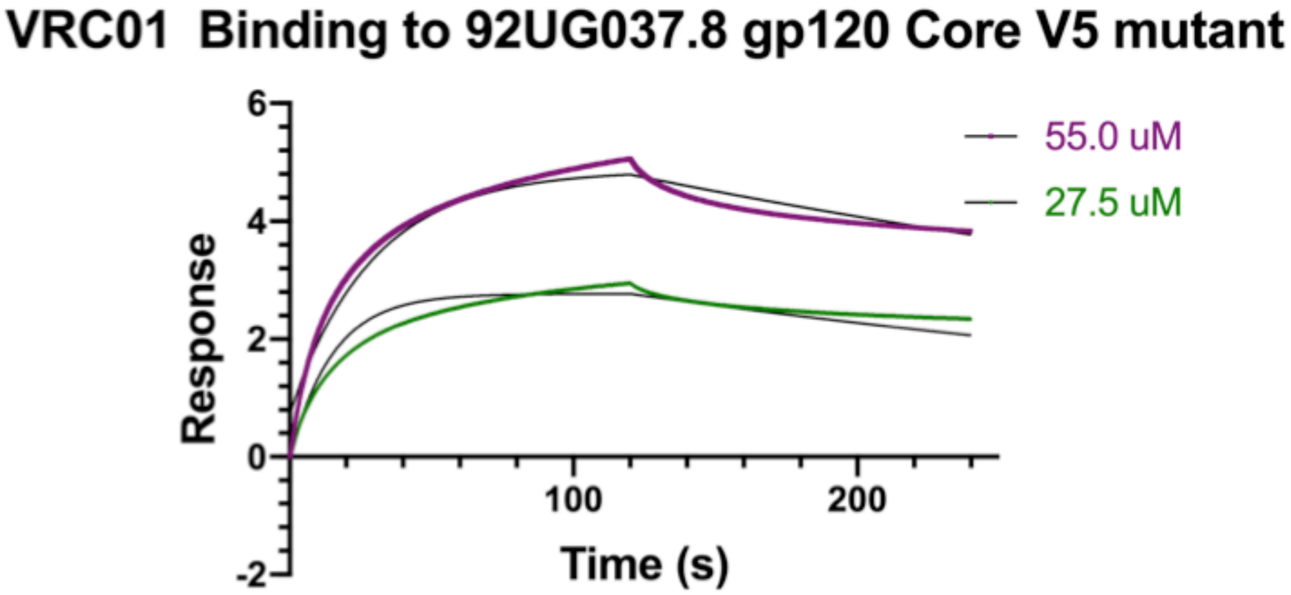

Supplementary Figure 2. Representative curves from biolayer interferometry for a non-CH103 lineage CD4bs bnAb, VRC01. The VRC01 Fab was immobilized onto an anti-human Fab-CH1 biosensor, and gp120 core constructs were introduced at three or more different concentrations, ranging from low micromolar to high micromolar. Fits and affinities derived as described in Methods.

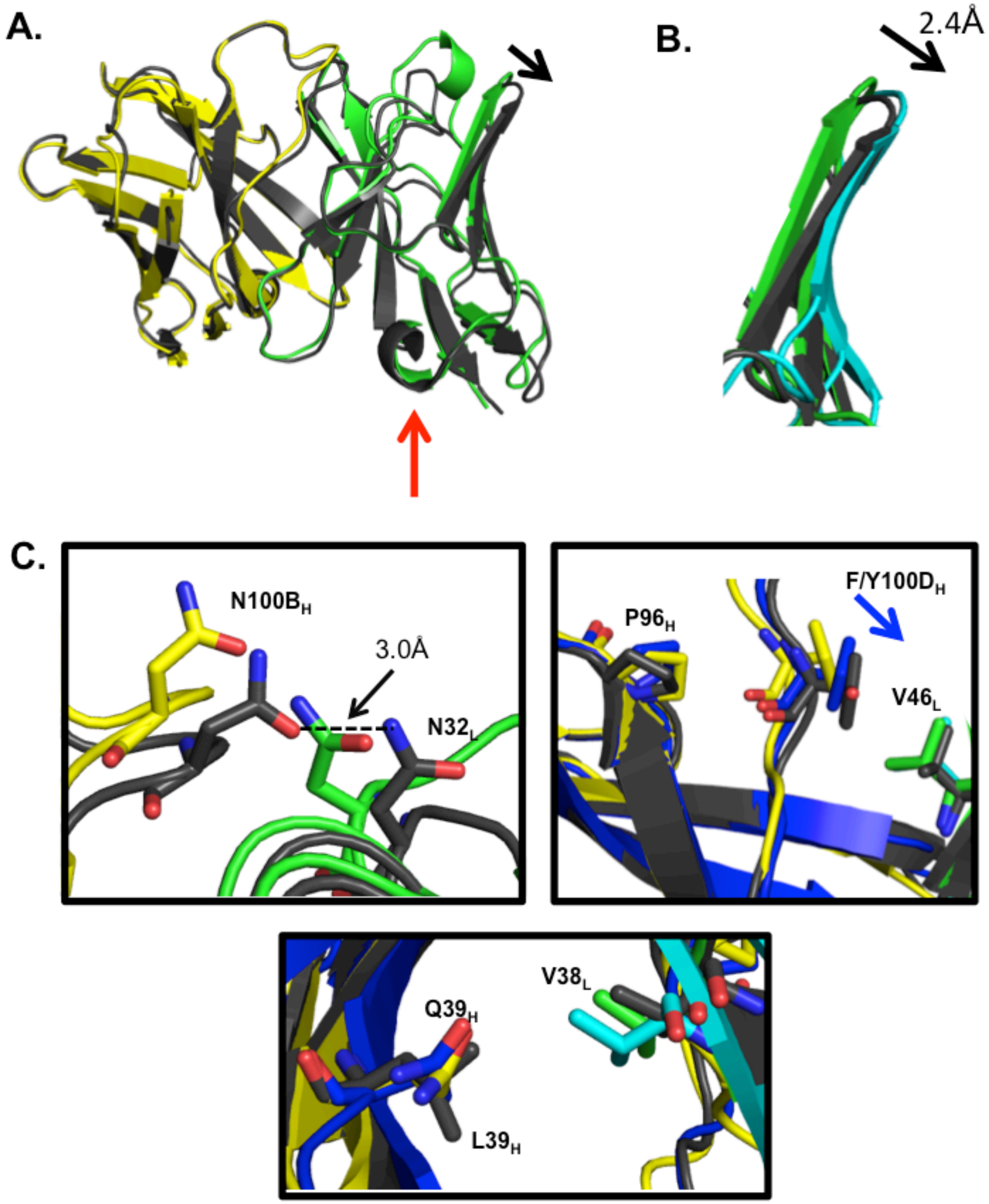

Supplementary Figure 3. Superpositions of mutant I33M, chimeric I3.1, and the CH103 bnAb. (A) Overall superposition of mutant Fab I33M onto the CH103 bnAb. The domains are colored: I33M V_H (yellow) and V_L (green), and CH103 (gray). Black arrows indicate regions where there is a conformational shift. Red arrow indicates the absence of a shift. (B) Zoomed in view of the DE loops of I33M (green), I3.1 (cyan), and CH103 (gray). The degree of shift is given. (C) Zoomed in views of residues that were mutated are shown as sticks for the three Fabs. Domains for the different Fabs are colored as in (A) and (B). I3.1 is excluded from the top left panel for clarity. Hydrogen bond distances are given.
